# Regulation of the p53 Family Proteins by the Ubiquitin Proteasomal Pathway

**DOI:** 10.3390/ijms21010261

**Published:** 2019-12-30

**Authors:** Scott Bang, Sandeep Kaur, Manabu Kurokawa

**Affiliations:** Department of Biological Sciences, Kent State University, Kent, OH 44242, USA; sbang8936@gmail.com (S.B.); skaur10@kent.edu (S.K.)

**Keywords:** apoptosis, cancer, Tp53, Tp63, Tp73, ubiquitination, E3 ligase

## Abstract

The tumor suppressor p53 and its homologues, p63 and p73, play a pivotal role in the regulation of the DNA damage response, cellular homeostasis, development, aging, and metabolism. A number of mouse studies have shown that a genetic defect in the p53 family could lead to spontaneous tumor development, embryonic lethality, or severe tissue abnormality, indicating that the activity of the p53 family must be tightly regulated to maintain normal cellular functions. While the p53 family members are regulated at the level of gene expression as well as post-translational modification, they are also controlled at the level of protein stability through the ubiquitin proteasomal pathway. Over the last 20 years, many ubiquitin E3 ligases have been discovered that directly promote protein degradation of p53, p63, and p73 in vitro and in vivo. Here, we provide an overview of such E3 ligases and discuss their roles and functions.

## 1. Introduction

The “guardian of the genome”, p53, has long been known to regulate the cellular responses of DNA repair, cell senescence, cell cycle arrest, and apoptosis [1]. Mice deficient in p53 exhibit significantly increased susceptibility to tumor formation compared to wild type mice and are a valuable tool with which to study the effects of p53 on tumor initiation and progression [2]. The importance of p53 is highlighted in the field of cancer by the fact that p53 is genetically deleted or mutated in approximately 50% of human cancers. Furthermore, in cancers that still maintain wild type p53, the signaling pathways upstream or downstream of p53 activation are often inhibited or rendered deficient by various mechanisms [3,4]. Mutant p53 has been shown to aid in tumor formation and progression not only by exhibiting a dominant negative inhibition of wild type p53, but also by showing oncogenic functions (gain of function) [5,6,7]. p53 is an extremely unstable protein and is known to be maintained at low levels in unperturbed conditions but is rapidly stabilized in response to various cellular stresses [8,9,10,11]. In contrast to wild type p53, mutant p53 is often stabilized and accumulates in cancer cells, allowing mutant p53 to exhibit its gain of function activity [5,6,7,11]. Our understanding of p53’s tumor suppressor function has recently expanded in scope, as p53 has been shown to respond to and activate numerous other cellular responses, including autophagy, metabolic reprogramming, stemness, tumor microenvironment signaling, and invasion and metastasis [12]. In addition, the role of “everyday” p53 has been gaining attention to highlight its role beyond causing tumor suppression, as p53 has been shown to regulate key homeostatic processes, such as metabolism, stem cell development and differentiation, and aging [13,14]. As such, the tight regulation of p53, at basal levels and in response to stress, is critical for p53 to exert its function when necessary [15,16].

The role of p63 and p73, the p53 family members, adds another layer of complexity to an already intricate system that remains to be fully fleshed out. p63 and p73 were initially discovered as homologues of p53 in 1998 and 1997, respectively, and were thought to have similar functions to p53 [17,18,19,20]. p53, p63, and p73 share many structural similarities, especially in the DNA binding domain and the transactivation domains [19,21,22,23]. They also share many of the same target genes. A key difference in p63 and p73 from p53 is the presence of two promoter regions that allow for the generation of isoforms that can be separated into two groups, namely, TAp63 and TAp73 isoforms that contain the N terminal transactivation domain and ∆Np63 and ∆Np73 isoforms that do not contain the N terminal transactivation domain [21,24,25]. Due to the presence of two promoters and alternative splicing that can generate several splice variants, the role of specific p63 and p73 isoforms in the context of cancer is quite complex. Interestingly, p63 and p73 mutations in human cancers are extremely rare, and expression of p63 and p73 variants differs widely depending on the type of cancer [23,26]. The TA isoforms are able to trigger apoptosis by inducing pro-apoptotic target genes shared with p53, pointing to tumor suppressor function, while the ∆N isoforms can act as dominant negative inhibitors of p53 and the TA isoforms of p63 and p73, suggesting an oncogenic role [23,27,28,29]. Unlike p53 knockout (KO) mice, p63 KO mice are embryonically lethal due to severe developmental deficiencies. Likewise, p73 KO mice show neurological defects but no increased rates of tumorigenesis as seen in p53 KO mice [30,31,32,33,34]. However, removal of just one copy of p63 or p73 (p63^+/−^ or p73^+/−^) makes the mice susceptible to spontaneous tumors, and mice heterozygous for deletions in p53 and p63 (p53^+/−^; p63^+/−^) or p53 and p73 (p53^+/−^; p73^+/−^) are both more prone to developing tumors compared to mice heterozygous for mutations in only p53 (p53^+/−^), suggesting that p63 and p73 do play a role in tumorigenesis [35]. Along the same lines, TAp73 KO mice have been shown to be more susceptible to spontaneous and stress-induced carcinogenesis [36]. As both p63 and p73 have been shown to be prognostic markers for certain types of cancer, the regulation of p63 and p73 and the interplay between its isoforms appear critical in understanding the role of the p53 network in tumorigenesis [23,26].

## 2. E3 Ligases for p53

p53 consists of multiple domains. Starting from the N terminus, it contains two transactivation domains, TAD1 (residues 1–40) and TAD2 (residues 41–61), followed by a proline rich domain (residues 63–97), then a highly conserved DNA binding domain (residues 102–192), and the tetramerization domain (residues 323–356) and regulatory domain (residues 363–393) comprise the rest of the p53 protein [37]. p53 is functionally active as a homo-tetramer and is able to bind and induce transcription of its target genes [38,39,40]. While p53 activity is controlled by post-translational modification of the protein, such as phosphorylation and acetylation, the abundance of p53 protein is strictly regulated by the ubiquitin proteasomal pathway. Under unstressed conditions, the half-life of p53 protein is 5–20 min [15,41,42,43]. In response to DNA damage, for instance, p53 protein becomes suddenly stabilized, resulting in the rapid accumulation of the protein and the robust induction of p53-target genes. Thus far, nearly 20 E3 ligases have been identified that target p53 for proteasomal degradation (for the full list of the p53-targeting ubiquitin E3 ligases, see Table 1) and additional non-E3 ligase proteins that also promote the degradation of p53 proteins (see Table 2).

### 2.1. MDM2/MDMX

**Figure 1:**
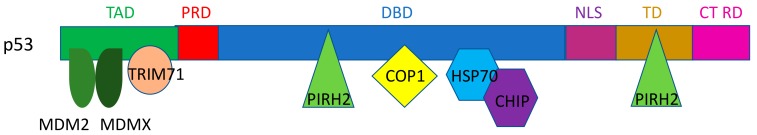
Schematic representation of interaction of p53 with different E3 ligases. Legend: TAD, transactivation domain; PRD, proline rich domain; DBD, DNA binding domain; NLS, nuclear localization signal; TD, tetramerization domain; CT RD, C terminal regulatory domain.

The primary regulator of p53 protein stability is the E3 ubiquitin ligase mouse double minute 2 (MDM2). MDM2 was first discovered as a gene that was amplified in the spontaneously transformed mouse 3T3-DM cell line [159]. Subsequently, human MDM2 was cloned and also found to be amplified in sarcomas, suggesting its potential role as an oncogene [46]. Two studies independently showed that MDM2 is able to bind p53 and inactivate its tumor suppressor function, indicating the tumorigenic potential of cells overexpressing MDM2 [56,160]. MDM2 consists of several domains: a p53-binding domain, a nuclear localization signal (NLS), a nuclear export signal (NES), a central acidic domain, a zinc-finger domain, and a RING-finger domain containing a nucleolar localization signal sequence. MDM2 can inhibit p53 through two mechanisms; it can promote proteasomal degradation of p53 and physically bind p53 to prevent its transcriptional activation [8,56,57,161,162]. MDM2 interacts with p53 via its N-terminal domain, binding to the transactivation domain of p53 and inhibiting its function [56,57] (Figure 1). The RING-finger domain and zinc-finger domain are required for MDM2 to exert its E3 ligase activity and ubiquitinate p53 for proteasomal degradation [163]. Depending on the levels of MDM2, MDM2 is able to dictate different fates for p53; low levels of MDM2 catalyze monoubiquitination of p53, causing its translocation to the cytoplasm, while high levels of MDM2 catalyze polyubiquitination of p53 and its subsequent degradation [164]. The importance of MDM2 as the primary regulator of p53 is highlighted by the embryonic lethality of MDM2 KO mice due to excessive p53 activation, which is able to be rescued by concomitant deletion of p53 [44,45]. Notably, as seen in some other p53-targeting E3 ligases (see below), MDM2 and p53 operate in a negative feedback loop, with p53 inducing the expression of MDM2, the very protein that works to degrade p53 [165,166,167].

MDMX (also known as MDM4), an MDM2 homolog, works to stabilize MDM2 through heterodimerization, preventing both MDM2 auto-ubiquitination and enhancing MDM2’s E3 ligase activity [168,169,170]. Although MDMX alone does not have significant E3 ligase activity, the gene KO of MDMX results in embryonic death due to lethal p53 activation [58,59,60]. As with the case of MDM2 KO, the lethal phenotype of MDMX KO can be fully rescued by co-deletion of the p53 gene. These results indicate that despite the lack of E3 activity, MDMX is as important as MDM2 in suppressing p53 activity. Notably, mutant mice deficient in MDM2–MDMX heterodimerization are also embryonically lethal, which can be rescued by concomitant deletion of p53 [171,172,173]. Thus, it is suggested that MDM2 and MDMX work together to inhibit p53, at least during embryo development. Interestingly, it is the MDM2–MDMX heterodimer that is essential in suppressing p53 during embryogenesis, rather than MDM2’s E3 ligase activity, and MDM2 E3 ligase activity is dispensable for development [171,174]. Taken together, these results suggest that MDM2 and MDMX function as a heterodimer, which not only promotes degradation of p53 protein, but also physically inhibits the function of p53 as a transcription factor independently of the ubiquitin proteasomal pathway.

Given that the MDM2–MDMX heterodimer plays the major role in the suppression of p53 activity and that genotoxic stress stabilizes p53 by releasing it from proteasomal degradation, one of the key questions would be what inhibits MDM2 and MDMX in response to genotoxic stress. An earlier study showed that DNA damage promotes degradation of MDM2 via self-ubiquitination [175]. However, recent studies using transgenic mice have demonstrated that catalytically inactive mutants of MDM2 degrade at the same rate as wild type MDM2 following DNA damage, regardless of whether they interact with MDMX, strongly suggesting the presence of another ubiquitin E3 ligase that targets MDM2 for degradation in response to genotoxic stress [171,174]. Interestingly, in contrast to MDM2, MDMX is a relatively stable protein under unstressed conditions. Nevertheless, DNA damage rapidly promotes degradation of MDMX protein by its binding partner MDM2. It remains to be fully elucidated as to how MDM2 and MDMX are regulated under various stress conditions, which would, in turn, impact the stability and activity of p53.

Consistent with its role as the major negative regulator of p53, MDM2 is often overexpressed or amplified in cancers. MDM2 amplification is seen in a panel of cancers, including sarcomas, gliomas, lymphomas, leukemia, and others [46,47,48,49,50,51,52,53]. Likewise, transgenic mice overexpressing MDM2 are more prone to spontaneous tumor development, specifically sarcoma and lymphoma [54]. Interestingly, amplification of MDM2 expression and p53 loss are often mutually exclusive [51,55]. MDMX has also been observed to be overexpressed in a variety of cancers, with evidence for oncogenic function by dampening p53’s tumor suppressor capabilities [61,62]. However, mouse models have shown conflicting results: in one study regarding MDMX overexpression in transgenic mice, the mice were shown to be more prone to tumor development, whereas another study found MDMX overexpression caused embryonic lethality, with heterozygous mice no more susceptible to tumor development compared to control mice [176,177]. Lastly, accumulating evidence strongly suggests that MDM2 and MDMX have p53-independent roles in genomic instability as well as tumorigenesis [54,55,61,62]. Given their major role as suppressors of p53 protein, the contribution of the p53-independent functions of MDM2/MDMX on the initiation and progression of various types of cancer remains to be explored further. Regardless, the frequency with which aberrant MDM2/MDMX expression is seen in cancer points towards a definitive role for MDM2 and MDMX in tumorigenesis.

### 2.2. Pirh2

p53 induced with a RING-H2 domain protein (Pirh2) is an E3 ubiquitin ligase that is able to interact with p53 in vitro and in vivo independently of MDM2 [68]. There are at least five known isoforms of Pirh2 that arise due to alternative splicing, with Pirh2A (full length Pirh2) the only isoform able to act as an E3 ubiquitin ligase, as it contains the RING-H2 domain necessary for its E3 ligase activity [68,178]. Residues 120 to 137 of Pirh2 are required for binding to p53, and Pirh2 binds to the central domain of p53 in residues 82 to 292 [68] (Figure 1). The RING-H2 domain of Pirh2 is required for Pirh2 to degrade p53, but interestingly, the RING-H2 domain is dispensable in the binding of Pirh2 to p53 and suppressing its transcriptional activation [68]. Like MDM2, Pirh2 and p53 work in a negative feedback loop, with p53 able to induce expression of Pirh2 [68]. However, unlike MDM2 and MDMX, Pirh2 appears to be dispensable for development, as Pirh2 KO mice are both fertile and born at the expected Mendelian ratios, with no obvious developmental defects [64]. In unstressed conditions, Pirh2 KO does not have significant effects on p53 levels in both cells and mice [64]. However, upon ionizing irradiation, Pirh2 KO mice show increased apoptosis as well as elevated levels of p53 and its target genes in various tissues compared to their control counterparts, indicating that Pirh2 plays a role in suppressing excessive p53 activation in response to DNA damage [64]. In this regard, Pirh2 functions as an oncogene. Supporting this notion, Pirh2 overexpression has been reported in various cancer types, including lung, breast, and prostate cancers and hepatocellular carcinomas [179,180,181,182,183]. However, Pirh2 also plays a role in promoting the degradation of other proteins, including c-Myc oncoprotein [64]. Accordingly, Pirh2 KO mice are more susceptible to spontaneous tumorigenesis due to elevated c-Myc levels and show significantly reduced tumor-free survival compared to wild type mice. Moreover, the tumor susceptibility has been observed to be further exacerbated by co-deletion of p53 [64]. Reduced expression of Pirh2 has been shown to be associated with worse prognoses in patients with breast cancer, ovarian cancer, and squamous cell carcinomas [64]. Therefore, it is suggested that Pirh2 may act as an oncogene or a tumor suppressor, depending on the tissue or cancer type.

### 2.3. COP1

COP1 contains an N-terminal RING finger domain that has ubiquitin ligase activity and WD40 repeats in the carboxyl terminus [184]. COP1 is highly conserved and ubiquitously expressed in human tissues, and localizes to both the nucleus and cytoplasm [184]. COP1 has been found to ubiquitinate p53 and target it for proteasomal degradation independently of MDM2 and Pirh2 [76]. The RING domain is required for COP1 to suppress p53 activity and has been found to regulate p53 in both stressed and unstressed conditions by interacting with p53 through the DNA binding domain (Figure 1) [76]. Like MDM2 and Pirh2, COP1 is also a p53 inducible gene [76]. Silencing COP1 expression in hepatocellular carcinoma (HCC) cells and in mouse xenograft models using HCC cells inhibits cell proliferation and tumor growth, respectively, in a p53-dependent manner, highlighting COP1 as a potential therapeutic target [185]. However, COP1 is also known to specifically bind c-Jun, a proto-oncogene, and other members of the Jun family, serving to downregulate their expression [69,184]. Knocking out COP1 in mice leads to embryonic lethality, with all COP1 null embryos showing severe developmental deficiencies by E10.5, and none surviving past E12.5 [69]. Interestingly, none of the COP1 null embryos show significant increases in apoptotic markers, and cardiovascular defects have been considered the likely cause of death [69]. Mice with COP1 hypomorphic alleles have been generated, and no significant increases in the levels of p53 have been seen, nor in the levels of p53 target genes in COP1 hypomorphic MEFs in both stressed and unstressed conditions [69]. Concomitant deletion of p53 has also been found to be unable to rescue the embryonic lethality of COP1^hypo/−^ mice, and COP1 hypomorphic mice have been found to be more susceptible to developing tumors due to c-Jun stabilization [69]. Further complicating the role of COP1 function, COP1 is upregulated in certain types of cancers, while also being functionally lost in others, leading to conflicting results as to whether COP1 functions as an oncogene or tumor suppressor [70].

### 2.4. CHIP

The carboxyl terminus of the Hsc70 interacting protein (CHIP) is an E3 ligase able to target its substrates for proteasomal degradation by binding the C termini of Hsc70 and Hsp90 and mediating the ubiquitination of chaperone bound proteins, with the U-box domain providing E3 ligase activity [87]. Through cooperation with Hsc70, CHIP ubiquitinates and targets p53 for proteasomal degradation, and silencing CHIP in U2OS cells stabilizes p53 [87]. CHIP is also able to degrade mutant p53 in addition to wild type p53 [87,186]. CHIP KO mice develop normally but are highly sensitive to heat stress [84]. CHIP KO mice die soon after thermal challenge, which has been attributed to an impaired stress response, with CHIP KO mice showing evidence of heat stroke [84]. Interestingly, CHIP KO mice have shown significant signs of apoptosis in splenocytes and the small intestine, suggesting p53 as a possible reason for this heightened stress response [84].

### 2.5. HUWE1

HECT, UBA, and WWE domain containing E3 ubiquitin ligase 1 (HUWE1, also known as ARF-BP1, Mule, and LASU1) is a HECT E3 ubiquitin ligase ubiquitously expressed in normal tissues [96]. HUWE1 directly binds and ubiquitinates p53 to target it for proteasomal degradation, independently of MDM2 [96]. Silencing HUWE1 in U2OS cells by RNAi has been found to stabilize p53 and lead to p53-dependent apoptosis [96]. However, HUWE1 has since been found to regulate the stability of a variety of substrates, including the proto-oncogene Myc and the pro-survival BCL2 family protein Mcl-1 [89,90,91]. While some of HUWE1’s substrates are tumor suppressors, others are known to promote cell survival. It should be noted that conditional deletion of *Huwe1* in fertilized mouse eggs has been found to result in embryonic lethality at E14.5 with a marked increase in the levels of p53 and caspase activation, indicating the significant role of HUWE1 in suppressing p53 during embryogenesis [88]. Conditional *Huwe1* KO in pancreatic β-cells also results in elevated p53 levels in these cells [88,187]. Likewise, in a mouse model of Myc-driven B cell lymphomas, *Huwe1* depletion was found to stabilize p53 and induce p53-dependent apoptosis and growth suppression [92]. In an analysis of E3 ligase expression in various types of human cancer, HUWE1 was found to be aberrantly expressed in seven out of nine types of tumors, including lung, breast, and prostate cancers [93]. This is consistent with previous studies observing HUWE1 overexpression in primary tumor samples [94,95]. Interestingly, however, targeted deletion of *Huwe1* in some mouse tissues, including keratinocytes, male germ cells, and hematopoietic progenitor cells, has not been found to lead to noticeable p53 activation [90,188,189,190]. Thus, while promoting the degradation of p53, the role of HUWE1 in cell survival and apoptosis may be context-dependent or tissue-specific. The role of HUWE1 with regard to p53 activation remains to be fully elucidated.

### 2.6. TRIM Proteins (TRIM24, TRIM28, TRIM29, TRIM39, TRIM69, and TRIM71)

Tripartite-motif-containing proteins (TRIM) are a group of proteins that are important for a plethora of biological functions, including tumorigenesis, metabolism, autophagy, and immunity [147,191,192]. TRIM proteins are characterized by an N-terminal RING finger domain that has ubiquitin ligase activity, one or two B-box zinc finger domains, and a coiled coil region [147]. There are over 70 TRIM proteins, and many are implicated to play a role in carcinogenesis (for further review, see [147]). Here we describe the TRIM proteins that are known to promote the degradation of p53.

TRIM24 has been found to function as an E3 ligase for p53 both in vitro and in vivo [108]. Silencing TRIM24 in cells has been seen to lead to a rise in endogenous p53 levels, and upon subjecting cells to DNA damaging reagents, upregulation of p53 target genes has been observed [108]. TRIM24 KO mice are viable, but interestingly show increased susceptibility to developing hepatocellular carcinomas [104]. However, tumor progression has been found to be greatly slowed down by inhibiting retinoic acid signaling, which is independent of the p53 pathway, suggesting that this liver carcinogenesis in TRIM24 KO mice may be mediated by a substrate of TRIM24 other than p53 [104]. High levels of TRIM24 expression are also associated with poorer prognoses in patients with breast cancer [105,106], although whether this can be ascribed to p53 suppression by TRIM24 remains unclear, as TRIM24 interacts with estrogen receptors and activates downstream pathways [105,106,107].

TRIM28 is another TRIM protein that was previously found to regulate p53, though it requires MDM2 for this function [99]. TRIM28 is able to ubiquitinate p53 for degradation through its interaction with MDM2, and silencing TRIM28 in cells causes an increase in expression of p53 target genes [99,193]. In humans, TRIM28 is highly expressed in many cancers and is linked to poorer overall survival [98]. TRIM28 KO mice are embryonically lethal, although it remains to be confirmed if this is solely due to fatal p53 activation [97]. Interestingly, unlike inducible MDM2 KO [194], acute deletion of the *Trim28* gene in adult mice does not appear to cause any obvious defects [195].

TRIM29, because it does not contain a RING finger domain, likely does not have E3 ubiquitin ligase activity. However, TRIM29 regulates p53 via binding p53 and sequestering it out of the nucleus, and works in conjunction with Tip60, an acetyltransferase protein, to inhibit p53’s transcriptional activity [152,196]. In addition to suppressing p53, TRIM29 positively regulates canonical Wnt signaling, stabilizes c-Myc, and inhibits the tumor suppressor PTEN. Accordingly, TRIM29 is found to be overexpressed in many cancers [148].

TRIM39 is yet another TRIM protein that has been demonstrated to regulate p53 in vitro and in vivo independently of MDM2 [113]. Silencing TRIM39 in cells expressing wild type p53 has been shown to enhance the potency of treatment with nutlin-3a (a compound that inhibits the binding between MDM2 and p53), suggesting TRIM39 and MDM2 have distinct roles in p53 regulation [113,197].

TRIM69 has recently been found to regulate p53 in the context of cataractogenesis [130]. TRIM69 has been shown to be able to bind p53 and induce its ubiquitination [130]. In human lens epithelial cells, TRIM69 overexpression has been found to decrease p53 protein levels and cause reduced apoptosis in response to ultraviolet B irradiation, an environmental risk factor for cataract development [130]. Conversely, silencing TRIM69 has been observed to have the opposite to expected effect, with cells having increased p53 levels and increased levels of apoptosis in response to ultraviolet B irradiation [130]. In a TRIM69 KO mouse model, TRIM69 was shown to have protective effects in the hippocampus of mice after a high fat diet challenge by inhibiting apoptosis and inflammation [129].

Lastly, TRIM71 (also known as LIN41) has been demonstrated to regulate p53 during stem cell differentiation [117]. TRIM71 KO mice are embryonically lethal due to the failure of the neural tube to close during development [114,115]. A recent study has demonstrated that TRIM71-mediated regulation of p53 is likely critical in facilitating normal embryonic stem cell differentiation and neurogenesis [117]. TRIM71 has been shown to bind p53 through its NHL domain, and is able to ubiquitinate p53 in embryonic stem cells [117]. Loss of TRIM71 increases p53 protein levels and reduces ubiquitination of p53, while TRIM71 induction increases cell proliferation and reduces apoptosis in a p53-dependent manner [117]. Most importantly, TRIM71 KO embryos have shown significantly increased levels of p53 and caspase activation by E10.5 [117]. Of note, in ovarian cancer cells, TRIM71 has been found to bind and target mutant p53 for degradation and inhibit mutant p53 target gene activation [116]. In line with the in vitro evidence, TRIM71 has been shown to suppress growth of ovarian tumors in mouse xenograft models as well [116].

### 2.7. RING1

Ring finger protein 1 (RING1) is a part of the transcriptional repression complex 1 (PRC1), which plays an important role in regulating embryonic development, stem cells, and cell proliferation. RING1 has been identified as an E3 ubiquitin ligase for p53, as RING1 depletion has been found to result in stabilization of p53 in both stressed and unstressed conditions [118]. RING1 is able to bind and ubiquitinate p53 to target p53 for proteasomal degradation in vitro and in vivo, and knockdown of RING1 is able to attenuate the proliferation of HepG2 and HCT116 cells as well as HepG2 xenografts in a p53-dependent manner [118]. Interestingly, RING1 expression has been found to be upregulated in hepatocellular carcinoma tissues compared to adjacent normal tissues from cancer patients, and higher RING1 expression has been associated with poorer prognoses [118].

### 2.8. FBW7α

F-box and WD repeat domain-containing protein 7 (FBW7) is an E3 ligase that belongs to the SCF group of E3 ligases, which uses Cullin-1 as a scaffold and F-box proteins as substrate receptors [198]. FBXW7, the gene encoding FBW7, is frequently mutated in human cancers, which is unsurprising considering FBW7 regulates a variety of oncogenic substrates, including c-Myc, Notch, Cyclin E, and c-Jun [198]. As such, several therapies have been explored to either restore FBW7 activity in cancers with mutated FBW7 or inhibit the downstream oncoproteins regulated by FBW7 [198]. FBW7 has three separate isoforms, namely, the α, β, and γ isoforms, which each localize to different regions of the cell [198]. Recently, FBW7, specifically the α isoform, has been found to be able to target and ubiquitinate p53 for proteasomal degradation [120]. Much like MDM2, p53 is known to regulate FBW7 transcription, forming an auto-regulatory feedback loop, and this regulation is crucial for maintaining genomic stability and the prevention of various types of cancer [119,121,122,123,124,125,126]. FBW7 KO mice are embryonically lethal, although it is not known whether this is due to lethal activation of p53 [127]. The importance of FBW7 in p53 regulation still needs to be explored further, as mouse models and TCGA data analyses all point to FBW7 having a significant role as a tumor suppressor, mainly due to the vast number of oncogenic substrates FBW7 has been shown to regulate [127,128].

## 3. E3 Ligases for p63 and p73

p63 and p73 are structurally similar to p53. From the N terminus, p63 and p73 are comprised of a transactivation domain, a proline rich domain, a DNA binding domain, an oligomerization domain, a second transactivation domain, a sterile α motif domain, and a transactivation inhibition domain [23,26]. The main difference arises from the alternative promoter regions of p63 and p73, which generate the TA and ∆N isoforms, with the ∆N isoforms lacking the N terminal transactivation domain. Both TA and ∆N isoforms can also be subdivided into the α, β, and γ isoforms, with the α isoforms containing the sterile α motif domain, which is important for protein–protein interactions and development [22,23,26]. Despite the structural homology, p63 and p73 are more stable proteins compared to p53 [199]. Nevertheless, multiple lines of evidence indicate that they are tightly regulated at the level of protein stability. Indeed, several ubiquitin E3 ligases have been discovered for p63 and p73 (for the complete list of the p63- and p73-targeting ubiquitin E3 ligases, see Table 3 and Table 4, respectively).

### 3.1. NEDD4

**Figure 2:**
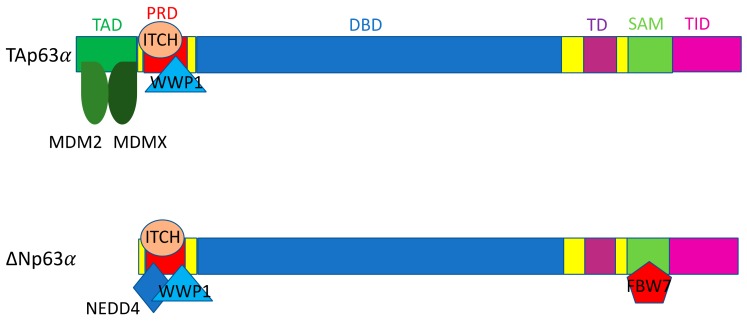
Schematic representation of interaction of p63 with different E3 ligases. Legend: SAM, sterile alpha domain; TID, trans inhibitory domain.

NEDD4 is an E3 ligase that contains a C terminal catalytic HECT domain and three WW domains that are required for protein–protein interaction [202]. NEDD4 targets ∆Np63 for proteasomal degradation through its interaction with the proline-rich domain of ∆Np63 (Figure 2) [203]. However, though p63 is a substrate of NEDD4, the importance of NEDD4 regulation of p63 in the context of cancer is overshadowed by the effects of NEDD4 regulation on its other substrates, most notably PTEN and c-Myc [202]. As such, both increased and decreased levels of NEDD4 are observed in different types of human cancer, making it likely that NEDD4’s role in tumorigenesis is variable depending on the context [202]. Of note, NEDD4 KO mice are embryonically lethal due to developmental heart defects, which is able to be mitigated through inhibition of thrombospondin-1 [201].

### 3.2. ITCH

**Figure 3:**
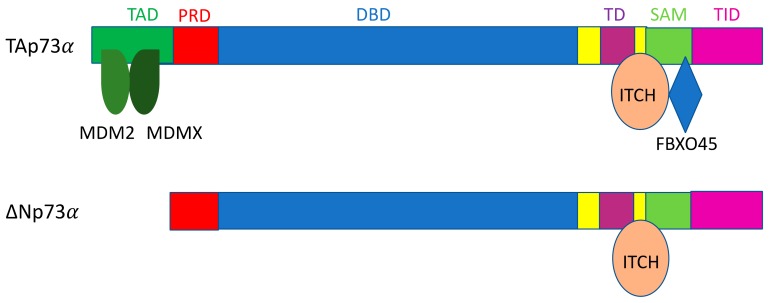
Schematic representation of interaction of p73 with different E3 ligases.

Itchy E3 ubiquitin protein ligase/atrophin-1 interacting protein 4 (ITCH/AIP4) is a HECT E3 ligase that can target both p63 and p73 for proteasomal degradation [206,207]. Interestingly, despite the structural similarity between p63 and p73, ITCH interacts with p63 and p73 through distinct domains/regions (Figure 2 and Figure 3) [206,207,219]. ITCH has also been shown to interact with MDM2 to target p73 for proteasomal degradation [220]. ITCH plays a role in a number of biological processes, including skin homeostasis and tumorigenesis, but is primarily a critical regulator of the immune response [221]. ITCH KO mice display severe immunological deficiencies, along with altered wound healing capability [204,221]. In lung cancer samples from patients, ITCH expression was found to be significantly upregulated, and depletion of ITCH inhibited cell proliferation and induced apoptosis in the lung cancer cells via the mitochondrial pathway [205]. Due to the diverse nature of substrates that ITCH regulates, however, the role of ITCH specifically in tumorigenesis still needs to be explored further.

### 3.3. WWP1

The WW domain containing E3 ubiquitin protein ligase 1 (WWP1) is an E3 ligase that contains a C terminal catalytic HECT domain and WW domains for protein–protein interaction, similarly to Pirh2 and NEDD4 [213]. WWP1 is able to both bind and ubiquitinate TAp63α and ∆Np63α for proteasomal degradation (Figure 2) [213]. In two non-tumorigenic breast epithelial cell lines, MCF10A and 184B5, WWP1 knockdown via siRNA caused an increase in ∆Np63α levels and conferred resistance to doxorubicin induced apoptosis in MCF10A cells [213]. On the flip side, in the HCT116 colon cancer cell line, stabilization of TAp63α was observed upon knocking down WWP1 via siRNA, and sensitized cells to both doxorubicin and cisplatin treatment in a p53-independent manner [213]. Given the fact that the ∆N isoforms of p63 can function as dominant negative inhibitors of p53 and the TA isoforms of p63, these results suggest that WWP1 may act as both an oncogene and tumor suppressor, at least in part, depending on the balance between ∆Np63 and TAp63 in the cells [213]. Following this line of reasoning, WWP1 is known to be overexpressed in types of prostate and breast cancer, but WWP1 also functions as an E3 ligase for several oncogenic substrates as well [212,233,234]. WWP1 has also been observed to heterodimerize with WWP2 to degrade ∆Np73 upon genotoxic stress, while WWP2 alone has been shown to be able to degrade full length p73 in unstressed conditions [230].

### 3.4. Pirh2

Pirh2, an E3 ligase that promotes the degradation of p53 (see above) can also target p63 and p73 for proteasomal degradation [235]. Pirh2 is able to ubiquitinate TAp63 and ∆Np63, which have been found to be vital for normal cell differentiation in the context of keratinocytes [218]. Pirh2 is also known to ubiquitinate p73 for degradation and has been found to specifically associate with the TAp73 isoforms [236,237]. Silencing Pirh2 in MCF7 cells significantly reduces cell proliferation and induces apoptosis in a TAp73-dependent manner [236]. In the context of cancer, Pirh2’s ability to promote p73 degradation appears to be important, considering that p73 is highly responsive to DNA damage like p53 [238]. Following DNA damage, Pirh2-mediated p73 ubiquitination and subsequent degradation are downregulated, allowing for p73 stabilization and p73-induced cell cycle arrest, independently of p53 [236,237]. 

### 3.5. MDM2/MDMX

The functional significance of MDM2 and MDMX as an E3 ligase for p63 and p73 remains unresolved and somewhat controversial, although a structural study has demonstrated that MDM2 can bind to the transactivation domains of all three p53 family proteins in vitro [200]. Early studies showed that MDM2 is able to interact with p73 but is unable to interact with p63 [239,240,241,242,243]. It has been noted, however, that despite its interaction with p73, MDM2 is not capable of inducing degradation of p73 [240,241,242]. Another study found that although MDM2 was unable to target p63 for degradation, it could rather hinder its transcriptional activity and inhibit p63-induced apoptosis, likely through exporting p63 out of the nucleus [244]. The nuclear export of p63 by MDM2 was confirmed later by another study; after nuclear export, FBW7 could then target p63, specifically the ∆Np63α isoform, for proteasomal degradation in the cytoplasm [217]. Of note is that other studies have shown that MDM2 is able to bind p63 and p73, but, interestingly, MDM2 interaction with p63 and p73 causes a stabilization of both proteins [245,246]. More recent literature is in agreement that MDM2 is able to bind both p63 and p73, but the interactions between MDM2 and p73 are much stronger than those between MDM2 and p63 [232,247]. In turn, because of the stronger interaction, MDM2 is much more effective in inhibiting the transcriptional activity of p73 isoforms compared to that of the p63 isoforms [232]. One study found that MDM2 is able to repress p73-mediated apoptosis and cell cycle arrest by ubiquitinating p73, and the RING domain of MDM2 is required for this activity [248]. Interestingly, overexpression of MDM2 is able to induce p73 degradation in conjunction with ITCH in MDM2-null MEFs, and a similar finding has previously been reported as well, which demonstrates that MDM2 is able to degrade p73 through interacting with ITCH in HeLa cells [220,248]. In p53-deficient lymphomas and sarcomas, the deletion of MDM2 could still induce apoptosis and cell cycle arrest through activation of p53 target genes, which were mediated by the stabilization of p73 [249]. Along the same lines, another study found that p73 deletion augmented the effects of MDM2 overexpression in the development of B-cell lymphomagenesis in mice, which was shown to promote genomic instability and tumor development [250].

Much like MDM2, early work showed that MDMX was able to interact with p73, but this interaction caused a stabilization of p73 [245]. Later, it was reported that MDMX, while able to interact with p73, was unable to interact with p63 and cause degradation of p63 protein [242,243], or that overexpression of MDMX was less effective in suppressing p63-induced apoptosis compared to that of MDM2, possibly due to MDMX being unable to export p63 out of the nucleus [244]. We should be aware that some of the early studies were conducted employing protein overexpression experiments rather than analyzing phenotypes at the endogenous protein levels, which might have resulted in contradictory results. Interestingly, biochemical analysis with recombinant proteins has demonstrated that MDMX is able to bind both p63 and p73 in vitro and that the interaction between MDMX and p73 is one order of magnitude stronger than the interaction between MDMX and p63 [247]. Moreover, the binding affinity of MDMX to p63 and p73 is stronger than that of MDM2 to p63 and p73 in vitro, suggesting that MDMX could play a larger role in regulating p63 and p73 compared to MDM2 [247]. Taken together, though MDM2 is well established as the master regulator of p53, further work needs to be done to understand the functional significance of MDM2/MDMX-mediated regulation of p63 and p73 protein stability.

## 4. Conclusions

Given p53’s firm role in the context of tumorigenesis, and now more recently in the context of metabolism, development, and homeostasis, it is clear that p53 is an indispensable aspect of our biology. p53 is tightly regulated at the protein level and kept at low levels in unstressed conditions, but quickly stabilized in response to stress. As such, it is vital to understand how exactly the E3 ligases regulate p53 in both stressed and unstressed conditions and the mechanism through which p53 stabilization occurs. In turn, the regulation of the E3 ligases themselves under both conditions needs to be fully elucidated as well. The matter is further complicated by the other p53 family members, p63 and p73. Despite the structural similarity between the p53 family members, they have varying E3 ligases that mediate their regulation, and each member performs exclusive functions in the cells that do not always overlap. Compared to p53, the significance of the specific E3 ligases in regulating p63 and p73 protein turnover, especially in the context of tumorigenesis, remains poorly understood. Due to the dynamic nature of p53 family member regulation and their functional overlap, the regulation of the p53 family members at the protein stability level needs to be explored further.

## Figures and Tables

**Table 1:** Ubiquitin E3 ligases That Directly Promote the Degradation of p53 Protein.

E3 Ligase	Phenotype of KO Mice	General Role in Cancer	p53 Interaction Site	Year ^#^
**MDM2**	Embryonically lethal [44,45]	p53 dependent and independent oncogenesis [46,47,48,49,50,51,52,53,54,55]	1–51 aa and C-terminus [56,57]	1997 [8]
**MDMX**	Embryonically lethal [58,59,60]	Oncogene [54,55,61,62]		2000 [63]
**PIRH2**	Predisposed to tumorigenesis [64]	Oncogene [65,66,67] Tumor Suppressor [64]	82–292 aa and the tetramerization domain [68]	2003 [68]
**COP1**	Embryonically lethal [69]	Oncogene [70,71,72,73] Tumor Suppressor [69,74]	Regions within the DNA-binding domain [75]	2004 [76]
**TOPORS**	Viable but high perinatal mortality, genomic instability [77]	Tumor Suppressor [77,78,79,80] Oncogene [81]		2004 [82]
**CHIP**	Sterile, high levels of protein oxidation and lipid peroxidation, reduced antioxidant defense system and energetic status [83], sensitive to heat stress [84]	Oncogene [85]	DNA binding domain in p53, Hsp70 and CHIP complex [86]	2005 [87]
**HUWE1 (ARF-BP1, MULE)**	Embryonically lethal [88]	Tumor suppressor [89,90,91] Oncogene [92,93,94,95]		2005 [96]
**TRIM28**	Embryonically lethal [97]	Oncogene [98]		2005 [99]
**CARP1/2**	CARP2 KO showed no abnormality [100]			2007 [101]
**SYNOVIOLIN**	Embryonically lethal [102]			2007 [103]
**TRIM 24**	Metastatic HCC [104]	Liver specific Tumor Suppressor [104] Oncogene in breast cancer [105,106,107]		2009 [108]
**JFK/SKP1-CUL1-F-box**		Oncogene in breast cancer [109]		2009 [110]
**MKRN1**		Senescence and tumorigenesis in gastric cancer [111]		2009 [112]
**TRIM 39**				2012 [113]
**TRIM 71**	Embryonically lethal [114,115]	Tumor suppressor by degrading mutant p53 [116]	TA domain of mutant p53 [116]	2017 [117]
**RING1**		Oncogene [118]		2018 [118]
**FBW7α**	Tumorigenic [119]	Oncogene [120] Tumor Suppressor [119,121,122,123,124,125,126,127,128]		2019 [120]
**TRIM 69**	Increased metabolic disorder after high fat diet treatment [129]			2019 [130]

^#^ Year p53 degradation activity was first discovered.

**Table 2:** Non-E3 ligases that Promote the Degradation of p53 Protein.

Name	Type	Phenotype of KO Mice	General Role in Cancer	Year *
**E6 HPV oncoprotein-E6AP complex**	Only in HPV infected cells	Viable but small prostate gland, increased p53 protein levels in prostrate [131]		1990 [132]
**P300/MDM2**	E4 ligase	Embryonic lethal [133]	Oncogene and tumor suppressor [134]	1998 [135]
**E4ORF6 and E1B55K**				2001 [136]
**ICP0**	HSV1 Viral Oncoprotein targeting p53 for translocation			2003 [137]
**UBC13**	E2 conjugating enzyme causing proteasome independent degradation- cytosolic localization, tetramerization inhibition	Embryonic lethal [138]	Oncogene [139,140]	2006 [141]
**E4F1**	An atypical E3 ligase (lacking HECT/RING), does not cause degradation or nuclear transport but carry out localization to chromatin	Embryonic lethal [142]	Oncogene [143,144]	2006 [145]
**TRIM 29**	TRIM protein lacking RING finger	Increased macrophage production [146]	Oncogene [147,148,149] Tumor Suppressor [150,151]	2010 [152]
**UBE4b**	E4 Ligase	Embryonic lethal [153]	Oncogene in breast cancer [154] Tumor suppressor [155,156]	2011 [157,158]

* Year p53 degradation activity was first discovered.

**Table 3:** Ubiquitin E3 Ligases for p63.

E3 Ligase	Isoform Specificity	Phenotype of KO Mice	General Role in Cancer	p63 Interaction Site	Year ^#^
**MDM2**	TAp63α * TAp63γ *	Embryonically lethal [44,45]	Oncogene [46,47,48,49,50,51,52,53,54,55]	TA Domain [200]	2001 [175] *
**MDMX**	TAp63α * TAp63γ *	Embryonically lethal [58,59,60]	Oncogene [54,55,61,62]		2001 [175] *
**NEDD4**	ΔNp63α	Embryonic lethality at mid gestation with heart defects [201]	Both [202]	PPPY motif in SAM domain [203]	2005 [203]
**ITCH**	ΔNp63α ΔNp63α	High rate of proliferation and improved wound healing [204]	Oncogene [205]	109-120 aa of TAp63 and 15-26 aa of ΔNp63 [206,207]	2006 [206,207]
**WWP1**	TAp63α ΔNp63α *	Increased rate of bone formation rates [208]	Oncogene in osteosarcoma [209], breast cancer [210,211], and prostate cancer [212]	PPPY motif in SAM domain [213]	2008 [213] 2010 [214]
**FBW7-MDM2**	ΔNp63α	Embryo lethality at day 10.5 due to defects in cardiovascular development [215,216]	Oncogene [120] Tumor Suppressor [119,121,122,123,124,125,126,127,128]	Region surrounding S383 [217]	2010 [217]
**PIRH2**	TAp63α ΔNp63α		Oncogene [67,71,72] Tumor Suppressor [64]		2013 [218]

^#^ Year p63 degradation activity was discovered. * Ubiquitination does not regulate protein stability but inhibits its transactivation activity.

**Table 4:** Ubiquitin E3 Ligases for p73.

E3 Ligase	Isoform Specificity	Phenotype of KO Mice	General Role in Cancer	p73 Interaction Site	Year ^#^
**MDM2**	TAp73α * TAp73β * ΔNp73α **	Embryonically lethal [44,45]	Oncogene [46,47,48,49,50,51,52,53,54,55]	TA Domain [200] SAM Domain [222]	1999 [170,171,172] *
**MDMX**	TAp73α * TAp73β *	Embryonically lethal [58,59,60]	Oncogene [54,55,61,62]		2001 [174] *
**ITCH**	TAp73α ΔNp73α	High rate of proliferation and improved wound healing [204]	Oncogene [205]	PY region just before the SAM domain of p73, and particularly the Y487 aa residue of TAp73 [219]	2005 [219]
**FBXO45**	TAp73α			SAM domain [223]	2009 [223]
**PIR2/RNF144B**	ΔNp73α		Oncogene [224]		2010 [225]
**PIRH 2**	TAp73α * TAp73β	Predisposed to tumorigenesis [64]	Oncogene [66,67,71] Tumor Suppressor [64]		2011 [166,167] *
**TRIM 32**	TAp73α	Myopathy and neurological deficiencies [226] AD- atopic dermatitis-like inflammatory skin condition [227]	Oncogene [228]		2013 [229]
**WWP2-WWP1 Complex**	ΔNp73α				2014 [230]
**HADES**					2015 [231]

^#^ Year p73 degradation activity was discovered. * Ubiquitination does not regulate protein stability but inhibits its transcriptional activity. ** ΔNp73α was also found to interact with MDM2 [232]. It remains to be determined which part of ΔNp73α MDM2 binds to as ΔNp73α does not contain the TA domain.

## Abbreviations

TAD	Transactivation domain
MDM2	Mouse double minute 2
Pirh2	p53 induced with a RING-H2 domain protein
CHIP	Carboxyl terminus of Hsc70 interacting protein
HUWE1	HECT, UBA, and WWE domain-containing E3 ubiquitin ligase 1
KO	Knockout
RING1	Ring finger protein 1
FBW7	F-box and WD repeat domain-containing protein 7
Itch/AIP4	Itchy E3 ubiquitin protein ligase/atrophin-1 interacting protein 4
WWP1	WW domain-containing E3 ubiquitin protein ligase 1
